# Effects of Hybrid Education Sequence on Learning Outcomes for Clinical Judgement Development in Nursing Students in the Developing Phase of Readiness

**DOI:** 10.31662/jmaj.2025-0211

**Published:** 2025-08-22

**Authors:** Ayako Nishimura, Yuma Ota, Yasuyo Kasahara

**Affiliations:** 1 Faculty of Healthcare, Division of Nursing, Tokyo Healthcare University, Tokyo, Japan

**Keywords:** nursing students, clinical judgment, competency-based education (CBE), simulation-based learning (SBL), educational design

## Abstract

**Introduction:**

This study aimed to determine the effects of the sequence of a hybrid class on learning outcomes for clinical judgment development in nursing students.

**Methods:**

This was a quasi-randomized controlled trial. The study participants consisted of 85 second-year nursing university students. The class was conducted in a hybrid format consisting of a simulation of providing nursing care to a patient with diabetes and an on-demand lecture on blood sugar regulation, insulin secretion, and action. The Japanese version of the Lasater Clinical Judgment Rubric (LCJR) was used to evaluate participants’ clinical judgment. The Shapiro-Wilk test confirmed non-normal distribution for all items. The Mann-Whitney U test compared groups with a significance level of p < 0.05. This study was based on the “Development and Validation of Guidelines for Reporting Evidence-Based Practice Educational Interventions and Teaching.”

**Results:**

No differences were found between the groups at baseline. No differences were found between the groups in achievement based on the LCJR items, total scores, or learning goals after the hybrid class.

**Conclusions:**

The results suggest that the sequence of simulations and on-demand lectures for clinical judgment development did not affect LCJR scores or learning goal achievement following a hybrid class.

## Introduction

Nurses’ clinical judgment can affect outcomes, such as failure to recognize a patient’s detrimental state change, failure to intervene, inappropriate management of complications ^(1)^, and errors in nursing diagnosis ^(2)^. Therefore, nursing education is expected to help students develop clinical reasoning for appropriate decision-making, clinical judgment based on decision-making ^(3)^, and safe and effective nursing practice skills that contribute to positive nursing outcomes through appropriate clinical judgment ^(4)^.

Clinical judgment is directly related to the quality of nursing and medical care, and strategies for teaching it include the transmission of information for situation assessment and judgment through dialogue between nursing faculty and students during clinical practicums, data selection of what is and is not important, and thought processes in setting actual nursing situations and evidence-based practice (EBP) ^(5)^. In particular, studies have reported that simulation learning is the most effective method for improving clinical judgment ^(6)^. High-quality simulation has been well documented as an intervention to develop clinical judgment competency in prelicensure nursing students ^(7)^. In a systematic review assessing the clinical reasoning of students and healthcare professionals ^(8)^, over half of the respondents reported using the Script Concordance Test or Lasater Clinical Judgment Rubric (LCJR) ^(9)^. The efficacy of various simulation methods, their impact on learning outcomes, and the reliability of associated indicators are currently being validated. In contrast, no reports are available on learning outcomes for comparing and sequencing different simulation formats to foster clinical judgment, in which EBP based on broad knowledge is essential.

Japanese nursing education needs to foster clinical judgment in practice and utilize clinical judgment models, such as the Clinical Judgment Model ^(10)^; however, other issues also need to be addressed, such as educational design and integration into the curriculum, given the overcrowded curriculum and various resource limitations, such as the number of simulators, simulated patients, classrooms, and faculty members. In recent years, the effectiveness of various teaching formats has been examined, including face-to-face and online education, educational methods, and communication technology utilization, in achieving optimal learning outcomes for nursing students ^(11)^. In particular, after the coronavirus disease 2019 pandemic, practice time and environment limitations have led to the consideration of hybrid teaching methods combining face-to-face and online instruction in competency-based education (CBE) and hybrid environments combining reality and augmented reality ^(12)^. Thus, the sequencing of simulations that foster effective and efficient clinical judgment, even with limited resources, urgently needs to be examined.

Therefore, this study aimed to clarify the impact of the sequence of a hybrid class (simulation and on-demand lectures) on the learning outcomes of nursing students in clinical judgment development.

## Material and Methods

### Study design and allocation

The study design was a quasi-randomized controlled clinical trial. The hybrid class (90 minutes in total) comprised a “simulation” (60 minutes) in which students practiced nursing care for a simulated patient with diabetes, including clinical judgment, and an “on-demand lecture” (30 minutes) on blood glucose regulation, insulin secretion, and action. The nursing students were divided into two groups (A and B). Group A attended an on-demand lecture after the simulation and Group B attended an on-demand lecture before the simulation. The results of the hybrid class were compared between Groups A and B. The students were assigned to the groups according to their student identification numbers. The educational design was based on CBE for clinical judgment development.

### Educational interventions

This study was based on the “Development and Validation of Guidelines for Reporting EBP Educational Interventions and Teaching” ^(13)^. The details of the applicable educational interventions are described in the subsections below.

#### Educational theories used in the educational intervention

In designing the hybrid class that served as the educational intervention in this study the preparation for the simulation was based on the National Council of State Boards of Nursing Simulation Guidelines for Prelicensure Nursing Education Programs ^(14)^. The simulation scenario development was based on the Healthcare Simulation Standards of Best Practice Simulation Design ^(15)^. Simulation design features, outcomes, facilitators, and educational practices were organized based on the National League for Nursing (NLN) Jeffries Simulation Framework ^(16)^, and a time schedule was created to secure time and opportunities for concrete experiences, reflective observations, and abstract concepts based on Kolb’s Experiential Learning Model ^(17)^, and a Simulation Research Rubric ^(18)^.

#### Hybrid teaching of clinical judgment courses that serve as educational interventions

This was a 1-credit (30 hours) course offered in the third semester of the student’s second year and a required “clinical judgment” course. The eight courses related to this study are on four specialized basic subjects, “Mechanism and Function of the Body,” “Pathophysiology,” “General Therapeutics,” and “Theory of Disease Treatment,” and four specialized subjects, “Assistive Technology for Daily Activities,” “Physical Assessment,” “Assistive Technology for Medical Treatment,” and “Critical Thinking and Nursing Processes.” This hybrid class builds on previously taken courses, and nursing students apply the knowledge, skills, and attitudes they have acquired to practice nursing, including clinical judgment.

### Learning goals of the hybrid class in CBE for clinical judgment development

The hybrid class in CBE for clinical judgment development had five learning goals. Students were given a pre-briefing 1 week prior to the start of the class, which included the learning goals, schedule, pre- and post-assignments, simulation learning, and preparation for on-demand lectures. The learning goals were as follows:

Learning Goal 1: [Preliminary Assignment] Able to gather appropriate information from electronic medical records.

Learning Goal 2: Able to explain the anatomy and physiology necessary for clinical judgment.

Learning Goal 3: Able to anticipate the patient’s condition, observe the condition with prioritization, and initially understand the condition.

Learning Goal 4: Able to interpret information from data obtained by observing the patient’s condition, respond as necessary, report using I-SBAR (Identify, Situation, Background, Assessment, and Recommendation) and nursing assistance.

Learning Goal 5: [Post-assignment] Able to reflect on the results and recognize the clinical judgment process.

#### Materials, preliminary assignments, and learning environment

A learning management system was used for the pre- and post-assignments in the hybrid class. The preliminary assignment was a case study to promote clinical judgment awareness presented using educational electronic medical records (Medi-EYE; Medi-LX Co., Ltd.), and students collected information. In addition, students were asked to collect and study the information necessary for the case using the Minds Clinical Practice Guidelines ^(19)^. The preliminary assignment was estimated to take approximately 2 hours, including the time necessary to collect information from the electronic medical records.

The simulations were conducted in an on-campus laboratory. A home-visit nursing bag was prepared for providing first-response care to a patient with diabetes. The bag contained a blood pressure cuff, stethoscope, SpO_2_(Peripheral capillary oxygen saturation) monitor, penlight, and stopwatch, as well as personal protective equipment. Simulations were performed with groups of five students and one faculty member serving as a facilitator, debriefer, and simulated patient for each group. One faculty member served as the overall facilitator for 30 students in total across six groups. The simulation was repeated four times by faculty members so that up to 120 students could participate.

#### Contents of EBP

Based on the Diabetes Mellitus Standard Medical Treatment Manual 2019 ^(20)^, the data needed to interpret the risks, benefits, and disadvantages ^(21)^ were included in the evidence and case scenarios incorporated into the decision support tool.

The students observed and initially responded to hypoglycemia in a simulated patient hospitalized for diabetes mellitus.

#### On-demand lecture details

The on-demand lecture was positioned within the hybrid class as preparatory or supplementary learning, depending on the group sequence. The lecture was designed by a faculty member specializing in physiology and endocrinology and lasted approximately 30 minutes. The objectives were to reinforce students’ understanding of blood glucose regulation, insulin secretion, and action in the context of diabetes care. The content included: (1) an overview of glucose homeostasis, (2) insulin physiology, (3) hypoglycemia/hyperglycemia mechanisms, and (4) clinical management. The lecture was developed in accordance with the Japanese Diabetes Society Guidelines (2019) and aligned with the learning goals of the hybrid class.

#### Simulation design developed as an educational intervention

The intervention in this study was a simulation design practice developed as an educational intervention. The simulations were designed by a faculty member who had received training, including the simulation educator step-up course “Applied Simulation Instructor Skills for Teaching (ASIST)” (co-sponsored by the University of Hawaii Simulation Center and JUNTENDO Medical Technology & Simulation Center) and had more than nine years of experience in simulation design, training instructors, and materials development. In addition, a pilot test was conducted by faculty members. The simulation was designed as CBE to enable students to acquire clinical judgment as a competency, defined as “an expected level of performance that integrates knowledge, skills, attitudes, and judgment” ^(22)^.

#### Scenario, simulation time schedule, and debriefing guideline

The scenario required nursing students to form multiple hypotheses of acute and chronic complications of hypoglycemia and hyperglycemia, make diagnostic judgments to determine the urgency and severity of hypoglycemic symptoms based on the rejection or adoption of the hypotheses, and make therapeutic judgments to consider safe and prompt intervention methods for hypoglycemic symptoms. The simulation time schedule is shown in Table 1. The Debriefing Guide (Table 1) is based on the framework of Tanner’s Clinical Judgment Model ^(10)^ of “awareness,” “interpretation,” “reaction,” and “reflection. The content was designed to elicit interpretation requiring analytical reasoning patterns based on previously taken subjects.

**Table 1.** Simulation Time Schedule.

Time	Task	Action
5 min	Orientation	Scenario theme・Learning objective・Briefing
		Pick students for simulation performance
5 min	Check pre-assignment	Gather information from electronic medical record and share for Learning objective 1
10 min	1st Simulation	1st performance for Learning objective 2
		The rest of the students take notes on white boards.
10 min	1st Debriefing	Perform based on anticipation and initial understanding
10 min	2nd Simulation	2nd performance for Learning objective 3
		The rest of the students take notes on white boards.
10 min	2nd Debriefing	Perform based on analytical inference pattern and action of clinical judgment
3 min	3rd Simulation	3rd performance for Learning objective 3
		The rest of the students take notes on white boards.
5 min	3rd Debriefing	Clinical judgment: Noticing, Interpreting, Responding, Reflection
2 min	Conclusion	Conclude toward learning objectives

### Subject of research

We conducted an educational intervention for 105 second-year students enrolled in the Department of Nursing at a university in one unit of the “Clinical Decision-Making” course, a hybrid class for the development of clinical decision-making. Overall, 85 students who provided informed consent were included in this study.

The sample size design was conducted using GPower 3.1.9.6, assuming a moderate effect size (f = 0.15) according to Cohen ^(23)^ and planning 77 students as the required sample size with a 5% significance level, 80% power, and effect size of 0.8.

### Study period

The study period lasted from December 2, 2021, to December 20, 2021.

### Data collection

Data were collected through a questionnaire administered at the end of each class. The researcher explained the purpose of the study orally and in writing to all students during an orientation 1 week prior to the class. All students were provided access to an electronic, anonymous, self-report questionnaire at the end of the class. Consenting students evaluated themselves and provided responses on their achievement levels in previously taken subjects, the LCJR ^(9)^, and their learning goals.

#### Achievement levels of eight previously taken subjects

Students self-evaluated using a 10-point Likert-type scale ranging from “achieved” to “not achieved” for four basic subjects: “Mechanism and Function of the Body,” “Pathophysiology,” “General Therapeutics,” and “Theory of Disease Treatment,” and four specialized subjects: “Assistive Technology for Daily Activities,” “Physical Assessment,” “Assistive Technology for Medical Treatment,” and “Critical Thinking and Nursing Process,” for eight subjects in total. This was used as a baseline measurement because it indicated the readiness levels of the participating students.

#### LCJR (11 items)

The LCJR is used in many countries to assess the development of learners’ clinical judgment ^(24)^. A Japanese version was developed previously and its reliability and validity have been verified ^(25)^. The Japanese version of the LCJR was used with permission to measure students’ clinical judgment.

The rubric has a total of 11 items across four categories and includes the following ratings: beginning (1 point), developing (2 points), accomplished (3 points), or exemplary (4 points). Overall scores ranged from 11 to 44.

・The noticing dimension emphasizes the ability to collect and recognize information.

・The interpreting dimension involves prioritizing relevant information and interpreting it to explain a patient’s condition.

・The responding dimension focuses on style habits, communication skills, intervention/flexibility, and the use of nursing skills.

・The reflecting dimension involves self-evaluation and commitment to improvement.

#### Achievement levels of learning goals for hybrid class in CBE for clinical judgment development

Students self-evaluated their achievement of the learning goals for the hybrid class using a 10-point Likert-type scale ranging from “achieved” to “not achieved” for Academic Goals 1, 2, 3, and 4.

### Analysis method

The sample size required for this study was calculated using G*Power based on the significance level, power, effect size, and number of independent variables. The significance level (α) was set at 0.05, power (1-β) at 0.80, effect size at 0.80, and allocation ratio N2/N1, and the sample size was calculated to be 52 participants.

The distribution of responses for all items on achievement levels in previously taken subjects, LCJR, and achievement of learning goals was found to confirm bias. The Shapiro-Wilk test was used to confirm that all items did not follow a normal distribution. The Mann-Whitney U test was used to compare groups, and the median and interquartile range values were listed. If an item had even one missing response, it was excluded from the dataset. Ultimately, 84 participants were included in the analysis.

All statistical analyses were performed using IBM SPSS version 27 (Japan International Business Machines Corporation, Tokyo, Japan) with a significance level of 5%. Two co-researchers reviewed all analyses to ensure analytical rigor.

### Ethical consideration

This study was approved by the Ethics Committee. Regarding the protection of the rights of the research participants, we assured them that their participation in the study was voluntary, they could withdraw their consent to participate even during the research period, and they would not suffer any academic disadvantages if they did withdraw. To protect the privacy of the participants, we explained that the survey responses were anonymous, the researchers could not know who had responded to the survey, and their grades would not be affected. Consent was checked using an electronic, anonymous, self-report questionnaire, and only the responses of those who provided consent were considered as data for the study. Non-participants were provided the same opportunities to participate in the classes and questionnaires as the participants. We also explained that the individual data and analysis results obtained would be used only for research purposes and the results would be submitted to an academic journal if their consent was provided.

To avoid any disadvantage to the learning outcomes as an ethical consideration, the presentation order was switched for the simulation and on-demand lecture for Groups A and B in the following class with a different case.

## Results

Overall, 84 responses were obtained for all the questions (valid response rate: 80.0%).

### Comparison of achievement in eight previously studied courses (baseline)

Participants’ achievement levels for the eight previously taken subjects were measured as an indicator of their readiness and compared between Groups A and B. The median scores for Anatomy and Physiology were 7.0 (Q1.5) in Group A and 6.0 (Q1.5) in Group B (p = 0.856), and for Pathophysiology, 6.0 (Q1.5) in both groups (p = 0.859), indicating no significant differences (Table 2).

**Table 2.** Comparison of Achievement in Eight Previously Studied Courses (Baseline Measurement).

			n = 84
	Group A	Group B	
Items	Median (quartile deviation)	Median (Q)	p value
Specialized Fundamental Courses			
Anatomy Physiology	M7.0 (Q1.5)	M6.0 (Q1.5)	0.856
Pathophysiology	M6.0 (Q1.5)	M6.0 (Q1.5)	0.859
General theory of therapeutics	M6.0 (Q1.0)	M6.0 (Q1.5)	0.96
Disease treatment	M6.0 (Q1.0)	M6.0 (Q1.5)	0.964
Specialized Courses			
Daily living assistance techniques	M7.0 (Q1.5)	M6.5 (Q1.5)	0.663
Physical Assessment	M6.5 (Q1.5)	M7.0 (Q1.5)	0.495
Assistive techniques for medical treatment	M6.0 (Q1.5)	M6.0 (Q1.0)	0.542
Critical thinking, Nursing process	M6.0 (Q1.5)	M6.0 (Q1.0)	0.301

*Mann-Whitney U test　*p<0.05,　**p<0.01　significance level 0.05 Comparison of achievement in eight previously studied courses (baseline measurement) between two groups in a hybrid class setting. The Mann-Whitney U test was performed to compare the two groups.

### Comparison of LCJR scores

A comparison of LCJR scores between Groups A and B revealed no significant differences for any items. For example, the total LCJR scores were 27.0 (Q4.5) in Group A and 27.0 (Q3.5) in Group B (p = 0.702), with similar trends across all subcategories (Table 3).

**Table 3.** Comparison of Lasater Clinical Judgment Rubric (LCJR) Scores.

			n = 84
Items	Group A	Group B	
	Median (quartile deviation)	Median (Q)	p value
Included in effective noticing: Subtotal	M8.0 (Q1.5)	M7.0 (Q1.0)	0.852
Focused observation	M3.0 (Q0.5)	M3.0 (Q0.5)	0.739
Recognition of deviation from expected patterns	M3.0 (Q0.5)	M2.5 (Q0.5)	0.675
Information searching	M3.0 (Q0.5)	M3.0 (Q0.5)	0.609
Included in effective interpreting: Subtotal	M5.0 (Q1.0)	M5.0 (Q1.0)	0.575
Prioritization of data	M3.0 (Q0.5)	M3.0 (Q0.5)	0.371
Making meaning of data	M2.5 (Q0.5)	M3.0 (Q0.5)	0.839
Included in effective responding: Subtotal	M9.5 (Q2.0)	M10.0 (Q1.5)	0.746
Calm and confident attitude	M2.0 (Q0.5)	M2.5 (Q0.5)	0.464
Clear communication	M3.0 (Q0.5)	M3.0 (Q0.5)	0.911
Well-planned intervention and flexibility	M2.5 (Q0.5)	M2.0 (Q0.5)	0.282
Being skilled	M2.0 (Q0.5)	M2.0 (Q0.5)	0.675
Included in effective reflection: Subtotal	M4.5 (Q1.0)	M5.0 (Q1.0)	0.906
Evaluation, self-analysis	M2.0 (Q0.5)	M2.0 (Q0.5)	1.749
Commitment to improvement	M2.0 (Q0.5)	M3.0 (Q0.5)	0.474
Rubric Total	M27.0 (Q4.5)	M27.0 (Q3.5)	0.702

*The Japanese version of the Lasater Clinical Judgment Rubric was used with permission from Dr. Hosoda. *Mann-Whitney U test　*p<0.05,　**p<0.01　significance level 0.05 Comparison of Lasater Clinical Judgment Rubric (LCJR) scores between two groups in a hybrid class setting. The Mann-Whitney U test was performed to compare the two groups.

### Comparison of achievement levels for learning goals in the hybrid class (Table 4)

The median achievement level of Learning Objective 1, which was the preliminary assignment, was median 7.0 for both groups, while the median achievement levels of Learning Objectives 2, 3, and 4, which were simulations, were median 6.0 for both groups. In Learning Objective 5, a median of 6.5 was observed in Group A and 6.0 in Group B, showing a difference between the groups, however, no significant difference was found. A comparison of the achievement levels of the learning goals after the hybrid class between Groups A and B showed no significant differences. For instance, Learning Objective 1 had a median score of 7.0 (Q1.0) in both groups (p = 0.95), and Learning Objectives 2 to 5 also showed median scores ranging from 6.0 to 6.5, with p-values above 0.05 (Table 4).

**Table 4.** Comparison of Achievement Levels for Learning Goals in the Hybrid Class in CBE.

			n = 84
	Group A	Group B	
	Median (quartile deviation)	Median (Q)	p value
Learning Objective 1【Pre-Assignment】	M7.0 (Q1.0)	M7.0 (Q1.0)	0.953
Able to gather appropriate information from electronic medical record.			
Learning Objective 2.	M6.0 (Q1.0)	M6.0 (Q1.0)	0.812
Able to explain the knowledge of anatomy and physiology necessary for clinical judgment.			
Learning Objective 3.	M6.0 (Q1.0)	M6.0 (Q1.0)	0.859
Able to anticipate the condition of the subject, observe the condition with prioritizing, and initially understand the condition.			
Learning Objective 4	M6.0 (Q1.0)	M6.0 (Q1.5)	0.086
Able to interpret information from data obtained by observing the patient’s condition and respond as necessary (I-SBAR reporting and nursing assistance).			
Learning Objective 5【Pre-Assignment】	M6.5 (Q1.0)	M6.0 (Q1.0)	0.305
Able to reflect on the results and recognize the clinical judgment process.			

Mann-Whitney U test　*p<0.05,　**p<0.01　significance level 0.05 Comparison of achievement levels for learning goals between two groups in the hybrid class in CBE. The Mann-Whitney U test was performed to compare the two groups.

## Discussion

### Simulation and lecture sequence in a hybrid class and learning outcomes

The purpose of this study was to determine the impact of the sequence used in a hybrid class (simulation and on-demand lecture) on learning outcomes for clinical judgment development in nursing students. The participating students were divided into two groups (A and B). Group A took part in the on-demand lecture after the simulation, whereas Group B participated in the simulation after the on-demand lecture. The results showed no differences in LCJR scores, which were used to measure nursing students’ clinical judgment after the hybrid lecture, or in their achievement of learning goals, suggesting no impact on learning outcomes. A previous study found that achievement levels in previously taken subjects, such as Physical Assessment and Anatomy and Physiology, influenced students’ ability to make clinical judgments. In this study, no differences were observed in achievement levels for previously taken subjects, which indicated the readiness of students in both groups. Thus, a hybrid class was designed to integrate the previously studied subjects based on the students’ readiness levels in those subjects. This suggests that even changing the order of the simulation and on-demand lecture in the hybrid class did not affect the learning outcomes. Students learned by relating the knowledge, skills, and attitudes they gained from previously studied subjects, which resulted in no differences in their clinical judgment levels or achievement of learning goals.

Students had already built a foundation for critical thinking, nursing processes, clinical reasoning, and clinical judgment, which correspond to basic assessment skills, through eight previously taken subjects. While on-demand lectures provide the advantage of flexibility―allowing students to review the content anytime and repeatedly―the sequence of simulation and lecture might still influence learning outcomes depending on the integration with simulation learning. Although both groups were in the developing phase and showed no significant differences in outcomes, further research should explore whether a unified sequence might promote educational fairness, or whether flexibility in sequence (with adequate support) can enhance individualized learning while maintaining consistent outcomes.

For simulation implementation, an effective and efficient education design is expected from the viewpoint of securing the number of simulators, number, and quality of simulated patients ^(26)^, cost of teaching materials ^(27)^, and digitalization of curricula ^(11), (18)^. However, in basic nursing education in Japan, including all students in the same year in one lecture, exercise, or simulation is difficult because of the ratio of faculty members to students and the classroom size. Consequently, teacher workloads are extremely heavy, such as conducting the same class multiple times in many basic nursing education programs. In addition, owing to the decline in learners’ readiness in primary and secondary education, decrease in enrollment, and overcrowded curriculum in basic nursing education, students are unable to retain and utilize knowledge unless they repeat.

Although the timetable for basic nursing education and the structure of the seminar room requires an educational design that repeats the same class multiple times, we believe it is significant that we could examine the content and evaluate the sequence of the hybrid class. The lack of differences in learning outcomes in the hybrid (distributed) class provides the effectiveness of educational design with limited resources.

Furthermore, no difference in learning outcomes in the sequence of the hybrid (distributed) class allows us to propose the educational design in CBE with limited resources. This study employed an educational design in CBE that integrated eight previously taken subjects related to clinical judgment and was expected to foster clinical judgment development. The results showed that Groups A and B were both “developing” in the four phases of clinical judgment; however, “developing” was considered to correspond to the level of achievement expected in undergraduate classes ^(25)^. Students had already built a foundation for critical thinking, nursing processes, clinical reasoning, and clinical judgment, which correspond to basic assessment skills, through eight previously taken subjects. Thus, few students were in the “rudimentary” phase, and over 50% were categorized as in the “developing” phase, which was the median value. In terms of learning goal achievement, both groups had a median score of 6.0 or higher for all learning goals, suggesting that they reached similar achievement levels in the subjects they had previously studied. This study’s instructional design was intended to help students integrate the content learned in the eight previously completed subjects. The hybrid class encouraged students to apply their prior knowledge to simulation-based practice. However, the questionnaire used in this study did not include a direct item asking students whether they were able to connect their previous knowledge to their current learning. This is an area for improvement in future research.

In this study, we set the competency and outcomes, and the educational design in the hybrid class in CBE to allow learning outcomes to be evaluated according to performance levels. In our research, we assert our innovative approach to enhancing clinical judgment in the hybrid class in CBE, optimizing nursing practice and outcomes through EBP. Based on the results of this study, it may be useful in future educational practice to adjust the sequence of simulations and on-demand lectures flexibly according to students’ readiness phases. For example, additional preparatory materials or follow-up learning opportunities could be provided to help students progressively develop clinical judgment skills.

### Limitations and future research directions

This was limited to a single university and the results were on the effects on learning outcomes of the sequence of simulations and on-demand lectures in a hybrid class using a single curriculum. Therefore, future studies need to survey multiple universities, and researchers should consider a systematic review of clinical judgment development in nursing education in Japan. In addition, since the data on achievement levels in previously studied subjects, LCJR scores, and learning goals were collected using students’ self-reports, the possibility of the Dunning-Kruger effect cannot be denied ^(28)^. Future consideration, including evaluation by others, is needed. Additionally, because this study relied on self-assessment data, there is a risk of response bias, including the Dunning-Kruger effect. For example, students with less developed clinical judgment skills might overestimate their abilities and report higher levels of achievement than is warranted. In addition, the learning outcomes in this study were measured by the LCJR with respect to clinical judgment immediately after the simulation and represent temporary and short-term results. In the future, it will be necessary to examine the long-term impact of clinical judgment, including the impact on other courses, timing of measurement, and post-course completion.

### Conclusion

The purpose of this study was to determine the effect of the sequence of a hybrid class (simulations and on-demand lectures) on learning outcomes for clinical judgment development in nursing students. The results suggest that in a hybrid (distributed) class, the sequence of simulations and on-demand lectures does not affect LCJR scores or the achievement of learning goals after the class. In the context of overcrowded curricula and the various resource limitations of simulators, simulated patients, classrooms, and faculty in basic nursing education in Japan, we believe it is significant that we could evaluate the sequence of the hybrid class for Clinical Judgment Development in CBE.

## Article Information

### Conflicts of Interest

None

### Author Contributions

Ayako Nishimura took the role of project administration acquiring funding and resources; conceptualized, designed, and conducted the study; designed the educational content; analyzed and interpreted the data including data curation; and prepared the manuscript. Yuma Ota designed the educational content; designed and conducted the study; analyzed and interpreted the data including data curation; and critically reviewed and edited the manuscript. Yasuyo Kasahara designed the educational content; designed and conducted the study; and critically reviewed and edited the manuscript.

### Approval by Institutional Review Board (IRB)

This study was approved by the Ethics Review Committee of Tokyo Healthcare University [Approval code: 32-43C].

### Informed Consent

Informed consent was obtained from all participants involved in this study.
